# Beyond smoking status: risk factors for pulmonary nodules ≥5 mm in a Chinese community screening program

**DOI:** 10.3389/fmed.2026.1857233

**Published:** 2026-07-22

**Authors:** Dong-mei Xue, Jing-lu Zhang, Zhi-lin Liu, Wen-hui Xiao, Yi-xin Zhou, Zhi-ye Zhang, Ke Lin, Ying Bian

**Affiliations:** 1 State Key Laboratory of Mechanism and Quality of Chinese Medicine, University of Macau, Taipa, Macau SAR, China; 2 Zhuhai Sanmed Biotech Ltd., Zhuhai, Guangdong, China; 3 Zhuhai Health Insurance Research Association, Zhuhai, Guangdong, China

**Keywords:** community screening, disease prevention, environmental health, pulmonary medicine, retrospective study

## Abstract

**Background:**

Community screening for pulmonary nodules in China primarily targets heavy smokers despite regional variation in smoking prevalence and environmental exposures. Few studies have assessed questionnaire-derived smoking and non-smoking factors in relation to low-dose computed tomography (LDCT) detected pulmonary nodules while accounting for smoking status.

**Objectives:**

To examine LDCT detected pulmonary nodules ≥5 mm across different smoking groups and to identify associated risk factors within high-risk individuals for lung cancer in a Chinese community screening cohort.

**Methods:**

This retrospective study analyzed data from a government-led community screening initiative in Zhuhai, China. Baseline screening records from 2021–2023 were used. High-risk individuals for lung cancer were identified using a standardized lung cancer risk-scoring system and advised to undergo low-dose computed tomography (LDCT). After data cleaning and restriction to participants with confirmed LDCT completion and uploaded LDCT findings, 5,414 participants were included. Multivariable logistic regression models assessed associations between demographic, smoking-related, lifestyle, environmental, familial, and clinical factors and LDCT detected pulmonary nodules ≥5 mm, overall and by smoking status.

**Results:**

Pulmonary nodules ≥5 mm were detected in 1,117 of 5,414 participants (20.6%). Detection was highest among never smokers (802/3,447, 23.3%), followed by former smokers (140/800, 17.5%) and current smokers (175/1,167, 15.0%). In the overall model, former smokers (aOR = 0.742, 95% CI: 0.593–0.928) and current smokers (aOR = 0.619, 95% CI: 0.503–0.762) had lower odds of nodules ≥5 mm than never smokers. Passive smoking exposure was associated with higher odds of nodules ≥5 mm (aOR = 1.257, 95% CI: 1.056–1.496), whereas irregular chest pain (aOR = 0.709, 95% CI: 0.573–0.877) and persistent cough (aOR = 0.806, 95% CI: 0.653–0.996) were associated with lower odds. Exploratory stratified analyses showed that subgroup-specific signals were mainly concentrated among never smokers.

**Conclusion:**

Among LDCT completed individuals at high risk for lung cancer, pulmonary nodules ≥5 mm were detected in one fifth of participants, especially among never smokers; passive smoking was positively associated with detection, while symptom and lung related history indicators showed inconsistent patterns. Community screening should strengthen risk assessment beyond active smoking, refine questionnaire-based indicators, and validate this strategy through multi region collaboration.

## 1 Introduction

Pulmonary nodules are among the most commonly detected abnormalities on chest imaging. They are regarded as potential indicators of lung cancer, the most common and deadliest cancer in China, with mortality nearly tripling from 1990 to 2022 (1, 2). Evidence shows that pulmonary nodules share key risk factors with lung cancer, including smoking, second-hand smoke exposure, indoor air pollution, dietary habits, and family history (3, 4). Among patients eventually diagnosed with lung cancer, 75.1% present with nodules ≥5 mm, and approximately 75% of nodules that progress to lung cancer are detected at stage I-II, with a curative rate of 77.9% following computed tomography (CT) or low-dose CT (LDCT) surveillance (5–8). Pulmonary nodules are also associated with a broad spectrum of pulmonary conditions beyond lung cancer, including infectious diseases, autoimmune and inflammatory disorders, vascular abnormalities, and congenital diseases (9, 10).

In China, pulmonary nodules are commonly detected through community-based lung cancer screening programs (11, 12). Clinical evaluation of pulmonary nodules often relies on size-based classification that reflects the increasing probability of clinically significant disease with larger nodule diameter. According to the *Chinese expert consensus on diagnosis and treatment of pulmonary nodules*, nodules are categorized as <5 mm (micronodules), 5–10 mm (small nodules), and >10 mm, the latter requiring prompt diagnostic evaluation and management (13).

However, the screening criteria are generally uniform nationwide and seldom adapted to regional risk patterns, typically targeting individuals aged ≥50 years with heavy smoking exposure (14, 15). In mainland China, environmental exposures and lifestyle patterns vary markedly across provinces, producing heterogeneous lung cancer risk profiles (16, 17). Typically, southern China bears the highest number of lung cancer cases, but its smoking prevalence is relatively low (≈20%) (18, 19). Guangdong, located in southern China and the most populous province nationwide, records the highest lung cancer mortality. In such settings, non-smoking-related factors, such as cooking-related exposures and preserved food intake, have been proposed as potentially relevant and warrant evaluation in local screening populations (20, 21).

Despite growing recognition of risk factors (22–24), community-based evidence remains limited on how various exposures contribute to screening-detected pulmonary nodules when smoking status is taken into account. This gap constrains the refinement of risk-adapted screening strategies, particularly in low-smoking settings. To address this, this study examined LDCT-detected pulmonary nodules ≥5 mm among high-risk participants for lung cancer who completed LDCT and assessed whether questionnaire-derived factors showed associations overall and in exploratory smoking-status-stratified analyses.

## 2 Methods

This retrospective study follows the Strengthening the Reporting of Observational Studies in Epidemiology (STROBE) guidelines (Supplementary material S2).

### 2.1 Setting and participants

Zhuhai is one of the nine core cities of the Guangdong-Hong Kong-Macao Greater Bay Area, with approximately 2.47 million residents (25). Data for this study were obtained from the Love Health (Ai Jian Kang) program, a government-led supplementary medical insurance screening initiative launched in 2020 by the Zhuhai Medical Insurance Bureau (26, 27).

The program focused on screening for ten major types of cancer including lung cancer. Participants were invited to take part through SMS messages, WeChat service accounts, and on-site promotional activities. Inclusion criteria: (i) be 18 years of age or older; (ii) have been enrolled in Zhuhai’s supplementary medical insurance for at least 1 year between 2021 and 2023; (iii) have lived locally for at least 6 months or have available medical insurance records in Zhuhai; (iv) be able to complete a structured questionnaire independently or with assistance; and (v) provide written or online informed consent. All participants completed a structured questionnaire developed for the program. The questionnaire captured sociodemographic characteristics, smoking-related factors, environmental and dietary exposures, lung-related history, family history, and recent symptoms (original version provided in Supplementary material S3).

Survey responses were anonymized by the Zhuhai Medical Insurance Bureau. High-risk individuals for lung cancer were identified using a standardized lung cancer risk-scoring system based on age, smoking exposure and other major risk factors, respiratory symptoms, and relevant medical history, with a total score ≥6 indicating high risk (Table 1). Since this scoring system was designed to identify individuals at high risk of lung cancer, self-reported history of pulmonary nodules was included as one of the high-risk criteria. However, to avoid conceptual overlap with the study outcome of LDCT-detected pulmonary nodules ≥5 mm, self-reported history of pulmonary nodules was not included as an independent variable in the subsequent regression models. Participants classified as high risk were advised to undergo LDCT and to upload their imaging results to the program platform. Only examinations conducted in public hospitals within the Zhuhai medical insurance system and meeting national standards were accepted (28).

**Table 1:** Criteria for high-risk classification of lung cancer.

Category	Criteria	Score^†^
Age	Age 40–49 years with smoking history	1
	Age ≥50 years with smoking history	2
Major risk factors	History of pulmonary nodules	6
	Cumulative smoking ≥10 years	2
	Average daily cigarette consumption: 5–20 cigarettes	1
	Average daily cigarette consumption ≥20 cigarettes	2
	Non-smoker with long-term passive smoking exposure	1
Clinical symptoms	History of chronic obstructive pulmonary disease (COPD) or diffuse pulmonary fibrosis	2
	Persistent cough for ≥1 month	1
	Hemoptysis within the past month	3
	Irregular chest pain persisting ≥1 month	2
Medical history	First-degree relative with lung cancer	2

^†^High-risk classification: Individuals with a total score of ≥6 are considered at high risk of lung cancer. The criteria were jointly developed by multiple experts in clinical medicine and public health, organized by the Zhuhai Health Insurance Research Association under the Zhuhai Medical Insurance Bureau.

### 2.2 Data cleaning and coding

**Figure 1:**
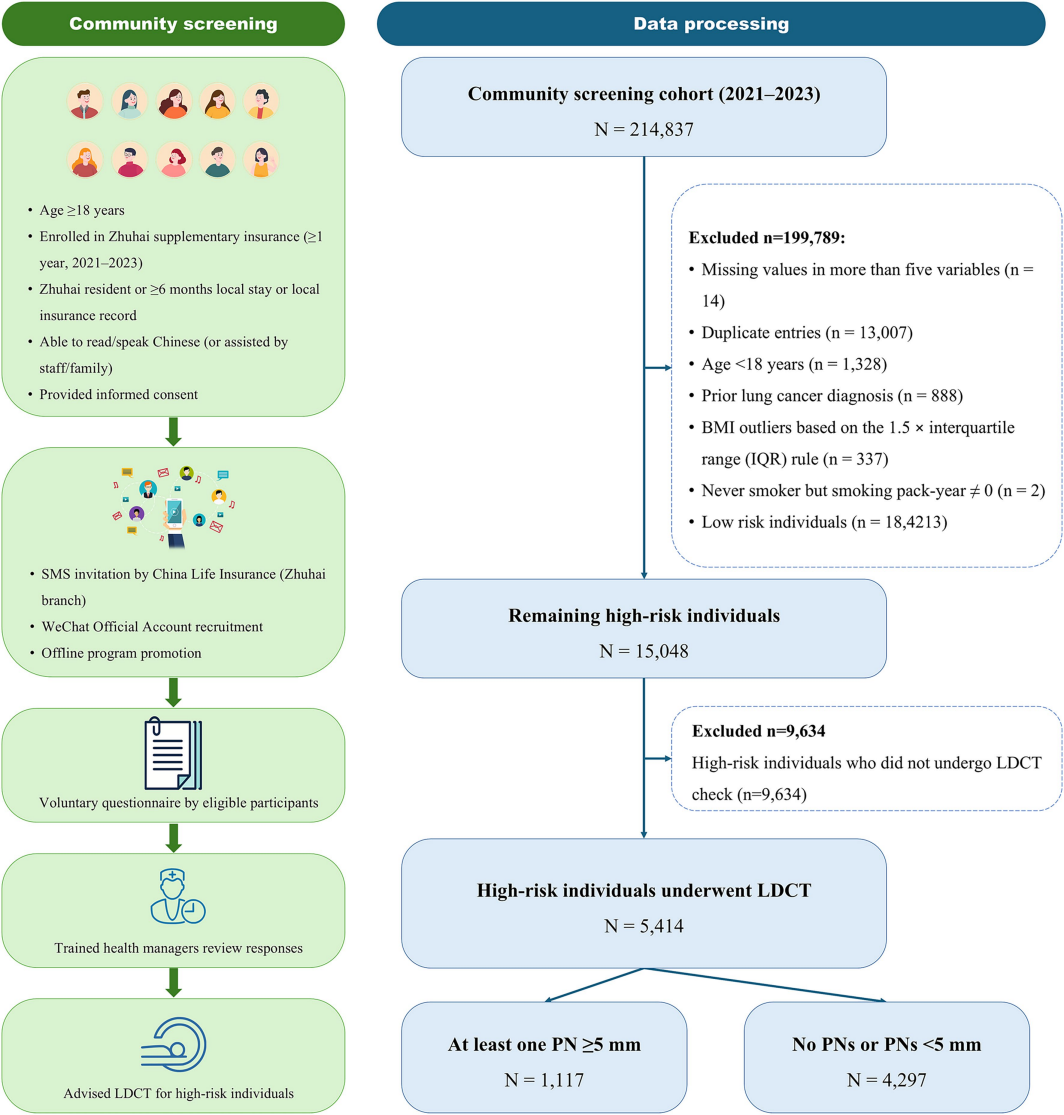
Study flowchart. BMI, body mass index; LDCT, low-dose computed tomography; PN, pulmonary nodules; SMS, short message service.

The community screening cohort included 214,837 questionnaire records collected between 1 January 2021 and 31 December 2023 (Figure 1). Before constructing the analytic sample, 199,789 records were excluded for the following reasons: missing values in more than five variables (*n* = 14), duplicate entries (*n* = 13,007), age <18 years (*n* = 1,328), prior lung cancer diagnosis (*n* = 888), BMI outliers identified using the 1.5 × interquartile range (IQR) rule (*n* = 337) (29), logically inconsistent smoking information, defined as never-smoking status combined with non-zero smoking pack-years (*n* = 2), and classification as low risk by the program’s lung cancer high-risk scoring system (*n* = 184,213). After these exclusions, 15,048 participants remained classified as high-risk individuals for lung cancer. To reduce outcome misclassification, high-risk individuals without confirmed LDCT examination were excluded from the pulmonary nodule analysis (*n* = 9,634) and were not classified as negative. The present analysis was further restricted to high-risk participants with confirmed LDCT completion and uploaded LDCT findings. Thus, the final analytic sample included 5,414 LDCT-completed high-risk participants. Among them, 1,117 participants had at least one reported pulmonary nodule ≥5 mm, whereas 4,297 participants had no nodules or only nodules <5 mm. After data cleaning and restriction to LDCT-completed participants, all variables included in the final models had no missing values, as shown in Supplementary Table S1.

The outcome variable was LDCT-detected pulmonary nodules ≥5 mm. It was coded as positive if the uploaded LDCT report indicated at least one pulmonary nodule with a diameter of ≥5 mm, and negative if the report indicated no nodules or nodules <5 mm only. Although follow-up imaging thresholds in many community-based lung cancer screening programs are commonly set at approximately 6–8 mm (30, 31), depending on nodule type and screening protocol, this program adopted a ≥5 mm operational threshold because the Chinese expert consensus classifies nodules measuring 5–10 mm as small pulmonary nodules (13). The ≥5 mm threshold was therefore used as the program-defined threshold for subsequent screening and management procedures.

Candidate independent variables were derived from the Love Health screening program questionnaire and were prespecified before model fitting. Variables were selected based on the program high-risk criteria, clinical relevance, and prior literature (Table 2). The final variables included sex assigned at birth (SEX), age (AGE), BMI, smoking status (SMK), smoking pack-years (SPY), passive smoking exposure (PS), history of chronic obstructive pulmonary disease (COPD), history of diffuse pulmonary fibrosis (DPF), irregular chest pain (ICP), hemoptysis (HEM), persistent cough (COU), hoarseness (HOA), cooking oil fume exposure (COOK), pickled or preserved food intake (FOOD), high salt or sugar intake (DIET), and first-degree relative history of lung cancer (FDR). Unexplained weight loss was once considered as a potential clinical symptom but was not included in the final model because no positive cases were recorded in the LDCT-completed analytic sample. Age, BMI, and smoking pack-years were retained as continuous variables. Smoking status was coded as a three-level categorical variable: never smoker, former smoker, and current smoker. Binary variables were coded as no or yes.

**Table 2:** Variables included in the model analysis.

Variable group	Variable	Definition	Data type	Basis for inclusion
Outcome	PN	LDCT-detected pulmonary nodules, defined as the presence of at least one reported pulmonary nodule with a diameter of ≥5 mm.	Binary	–
Demographic and anthropometric factors	SEX	Sex assigned at birth, categorized as male and female.	Binary	(72)
	AGE^†^	Age at questionnaire completion, measured in years.	Continuous	(13)
	BMI	Body mass index, calculated as weight in kilograms divided by height in meters squared (kg/m^2^).	Continuous	(73)
Smoking-related factors	SMK	Smoking status, categorized as never smoker, former smoker, and current smoker.	Categorical	(39, 40)
	SPY^†^	Smoking pack-years, calculated as (cigarettes per day ÷ 20) × years smoked; coded as 0 for never smokers.	Continuous	(39)
	PS^†^	Passive smoking exposure, defined as self-reported long-term exposure to second-hand smoke.	Binary	(40)
Lung-related history	COPD^†^	History of chronic obstructive pulmonary disease.	Binary	(59)
	DPF^†^	History of diffuse pulmonary fibrosis.	Binary	(74)
Clinical symptoms	ICP^†^	Irregular chest pain persisting for ≥1 month.	Binary	(45)
	HEM^†^	Hemoptysis reported in the past month.	Binary	(46)
	COU^†^	Persistent cough for ≥1 month.	Binary	(46)
	HOA	Hoarseness reported in the past month.	Binary	(61)
Household and dietary exposures	COOK	Cooking oil fume exposure, defined as frying, stir-frying, baking, or pan-frying ≥3 times per week.	Binary	(49)
	FOOD	Intake of pickled or preserved foods at least once per week.	Binary	(49)
	DIET	High salt or sugar intake, defined as daily salt intake >5 g or sugar intake >30 g.	Binary	(50, 51)
Family history	FDR^†^	First-degree relative history of lung cancer, defined as lung cancer history among parents, children, or siblings.	Binary	(75)

^†^Variables that were included in high-risk criteria for the screening program. AGE, age; BMI, body mass index; COOK, cooking oil fume exposure; COPD, chronic obstructive pulmonary disease; COU, persistent cough; DIET, high salt or sugar intake; DPF, diffuse pulmonary fibrosis; FDR, first-degree relative history of lung cancer; FOOD, pickled or preserved food intake; HEM, hemoptysis; HOA, hoarseness; ICP, irregular chest pain; LDCT, low-dose computed tomography; PN, pulmonary nodules ≥5 mm; PS, passive smoking exposure; SEX, sex assigned at birth; SMK, smoking status; SPY, smoking pack-years.

### 2.3 Statistical analysis

Continuous variables were evaluated for distributional characteristics using skewness, kurtosis, and percentile distributions (32). Since all continuous variables showed departures from normality, they were summarized as medians with IQRs. Categorical variables were summarized as numbers and percentages. Comparisons between participants with and without LDCT-detected pulmonary nodules ≥5 mm were performed using Mann–Whitney *U* tests for continuous variables and Pearson chi-square tests for categorical variables.

The primary analysis used multivariable logistic regression with the enter method to estimate associations between questionnaire-derived variables and LDCT-detected pulmonary nodules ≥5 mm. The presence of at least one pulmonary nodule ≥5 mm was specified as the dependent variable. Results were reported as adjusted odds ratios (aORs) with 95% confidence intervals (CIs). Model diagnostics were performed for the primary multivariable model. Multicollinearity was assessed using variance inflation factors (VIFs), with VIF < 10 considered acceptable (33). Model calibration and goodness-of-fit were assessed using the Hosmer-Lemeshow test. The number of events per predictor parameter and pseudo-*R*^2^ statistics were also reported to describe model stability and explanatory capacity.

To examine whether associations differed by smoking status, subgroup logistic analyses with enter method were conducted separately among never smokers, former smokers, and current smokers. Smoking status was not included as a covariate in subgroup-specific models. Smoking pack-years were not included in the never smoker model because they were coded as 0 for never smokers after logical consistency checks. Smoking pack-years were retained as a continuous covariate in the former smoker and current smoker models. Given the exploratory nature of subgroup analyses and the presence of multiple comparisons, subgroup-specific *p* values were additionally assessed using the Benjamini-Hochberg false discovery rate procedure.

Several sensitivity analyses were conducted to assess the robustness of the findings. On the one hand, backward likelihood ratio selection was used as an alternative modeling approach to examine whether the primary enter-method results were dependent on the variable entry strategy. On the other hand, sequential logistic regression models were fitted to examine whether the association between smoking status and pulmonary nodule detection could be explained by differences in age and sex distributions across smoking-status groups. These models included smoking status alone, smoking status adjusted for age and sex, smoking status further adjusted for BMI, smoking pack-years, and passive smoking, and the fully adjusted model. All analyses were conducted using SPSS version 27.0 (IBM Corp., Armonk, NY, United States). Two-sided *p*-values <0.05 were considered statistically significant in the primary analysis.

## 3 Results

### 3.1 Participant characteristics

After data cleaning and restriction to program-defined lung cancer high-risk participants with confirmed LDCT completion, the final analytic sample included 5,414 participants. Among them, 1,117 participants (20.6%) had at least one LDCT-detected pulmonary nodule ≥5 mm, whereas 4,297 participants (79.4%) had no nodules or only nodules <5 mm (Table 3). In the total analytic sample, the median age was 49 years (IQR, 15), the median BMI was 22.78 kg/m^2^ (IQR, 4.05), and the median smoking pack-years was 0.00 (IQR, 6.00) pack-years. Age and BMI were similar between participants with and without nodules ≥5 mm, with no statistically significant between-group differences. Smoking pack-years differed significantly by nodule status; although the median was 0.00 in both groups, the IQR was larger among participants without nodules ≥5 mm than among those with nodules ≥5 mm (7.50 vs. 0.63 pack-years; *p* < 0.001).

**Table 3:** Characteristics of LDCT-completed participants by pulmonary nodule status.

Variable	No nodules or nodules <5 mm (*n* = 4,297, 79.4%)	At least one nodule ≥5 mm (*n* = 1,117, 20.6%)	Total (*n* = 5,414, 100%)	*p*-value
Continuous variables, median (IQR)				
AGE, years	50 (14)	49 (15)	49 (15)	0.317
BMI, kg/m^2^	22.77 (4.09)	22.83 (3.84)	22.78 (4.05)	0.715
SPY, pack-years	0.00 (7.50)	0.00 (0.63)	0.00 (6.00)	<0.001***
Categorical variables, *n* (%)				
Sex				<0.001***
Male	2,082 (82.0)	457 (18.0)	2,539 (46.9)	
Female	2,215 (77.0)	660 (23.0)	2,875 (53.1)	
SMK				<0.001***
Never smoker	2,645 (76.7)	802 (23.3)	3,447 (63.7)	
Former smoker	660 (82.5)	140 (17.5)	800 (14.8)	
Current smoker	992 (85.0)	175 (15.0)	1,167 (21.6)	
PS				0.019*
No	918 (81.9)	203 (18.1)	1,121 (20.7)	
Yes	3,379 (78.7)	914 (21.3)	4,293 (79.3)	
COPD				0.259
No	4,170 (79.3)	1,091 (20.7)	5,261 (97.2)	
Yes	127 (83.0)	26 (17.0)	153 (2.8)	
DPF				0.063
No	4,118 (79.2)	1,084 (20.8)	5,202 (96.1)	
Yes	179 (84.4)	33 (15.6)	212 (3.9)	
ICP				<0.001***
No	3,638 (78.5)	995 (21.5)	4,633 (85.6)	
Yes	659 (84.4)	122 (15.6)	781 (14.4)	
HEM				0.238
No	4,160 (79.3)	1,089 (20.7)	5,249 (97.0)	
Yes	137 (83.0)	28 (17.0)	165 (3.0)	
COU				0.002**
No	3,627 (78.7)	984 (21.3)	4,611 (85.2)	
Yes	670 (83.4)	133 (16.6)	803 (14.8)	
HOA				0.338
No	3,944 (79.2)	1,035 (20.8)	4,979 (92.0)	
Yes	353 (81.1)	82 (18.9)	435 (8.0)	
COOK				0.533
No	382 (78.3)	106 (21.7)	488 (9.0)	
Yes	3,915 (79.5)	1,011 (20.5)	4,926 (91.0)	
FOOD				0.493
No	342 (80.7)	82 (19.3)	424 (7.8)	
Yes	3,955 (79.3)	1,035 (20.7)	4,990 (92.2)	
DIET				0.321
No	3,511 (79.1)	927 (20.9)	4,438 (82.0)	
Yes	786 (80.5)	190 (19.5)	976 (18.0)	
FDR				0.841
No	3,531 (79.4)	915 (20.6)	4,446 (82.1)	
Yes	766 (79.1)	202 (20.9)	968 (17.9)	

Continuous variables are presented as medians (IQRs) and were compared using Mann–Whitney *U* tests. Categorical variables are presented as *n* (%) and were compared using Pearson chi-square tests. Percentages in the pulmonary nodule status columns are row percentages, and percentages in the total column are percentages of the total analytic sample. *p < 0.05; **p < 0.01; ***p < 0.001. LDCT, low-dose computed tomography; AGE, age; BMI, body mass index; SMK, smoking status; SPY, smoking pack-years; PS, passive smoking exposure; COPD, chronic obstructive pulmonary disease; DPF, diffuse pulmonary fibrosis; ICP, irregular chest pain; HEM, hemoptysis; COU, persistent cough; HOA, hoarseness; COOK, cooking oil fume exposure; FOOD, pickled or preserved food intake; DIET, high salt or sugar intake; FDR, first-degree relative history of lung cancer.

Several participant characteristics and questionnaire-derived factors differed between participants with and without nodules ≥5 mm. Females had a higher crude proportion of nodules ≥5 mm than males: 660 of 2,875 females (23.0%) had at least one nodule ≥5 mm, compared with 457 of 2,539 males (18.0%; *p* < 0.001). Nodule detection also differed by smoking status and was highest among never smokers (802/3,447, 23.3%), followed by former smokers (140/800, 17.5%) and current smokers (175/1,167, 15.0%; p < 0.001). Participants with passive smoking exposure had a higher proportion of nodules ≥5 mm than those without passive smoking exposure (914/4,293, 21.3% vs. 203/1,121, 18.1%; *p* = 0.019). By contrast, nodule detection was lower among participants reporting irregular chest pain than among those without irregular chest pain (122/781, 15.6% vs. 995/4,633, 21.5%; *p* < 0.001). A similar pattern was observed for persistent cough, with a lower proportion of nodules ≥5 mm among participants reporting persistent cough than among those without persistent cough (133/803, 16.6% vs. 984/4,611, 21.3%; *p* = 0.002). No statistically significant crude differences by nodule status were observed for COPD, diffuse pulmonary fibrosis, hemoptysis, hoarseness, cooking oil fume exposure, pickled or preserved food intake, high salt or sugar intake, or first-degree relative history of lung cancer.

### 3.2 Factors associated with pulmonary nodules ≥5 mm in the total cohort

**Figure 2:**
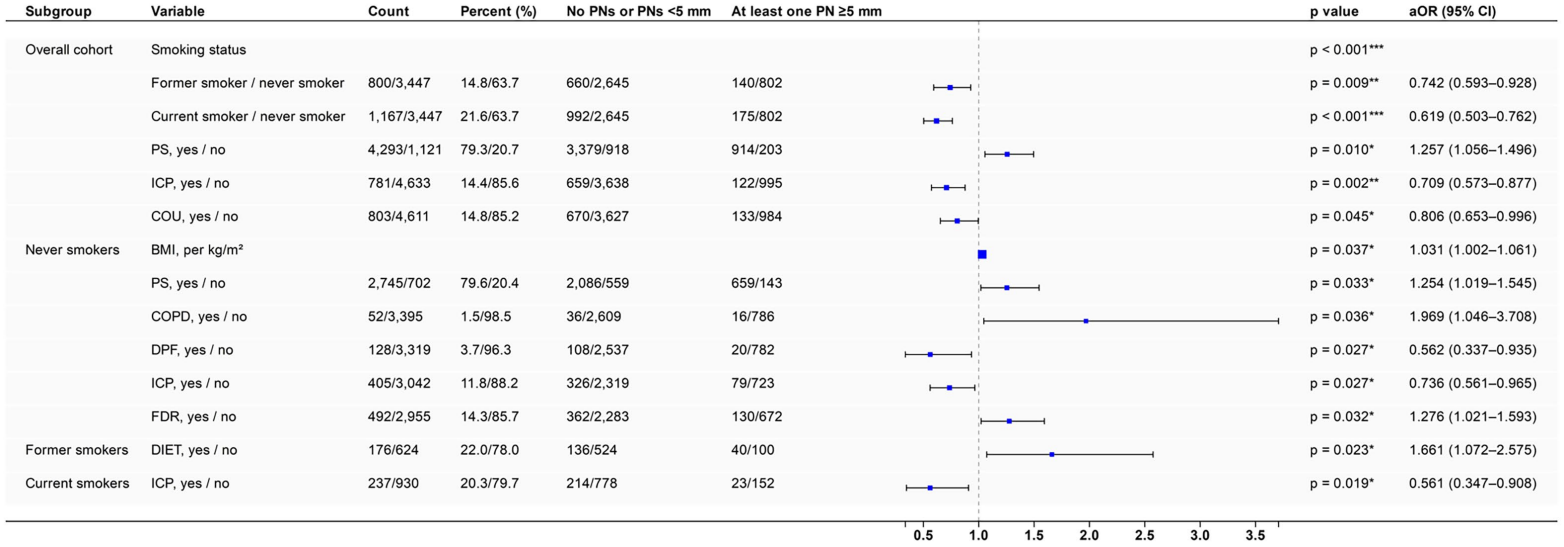
Overall and smoking-status-stratified significant associations with LDCT-detected pulmonary nodules ≥5 mm. Note. This figure displays variables that were statistically significant in the overall multivariable model and variables that were nominally significant in smoking-status-stratified models. Count and percent values are presented as comparison/reference groups for smoking status and as yes/no groups for binary predictors. Percentages were calculated within the corresponding analytic population: the overall cohort for overall-model estimates and the relevant smoking-status subgroup for stratified estimates. BMI was modeled as a continuous variable; therefore, count and percentage values are not shown. Asterisks indicate nominal statistical significance before multiple-comparison correction: *p < 0.05; **p < 0.01; ***p < 0.001. Subgroup-specific findings should be interpreted as exploratory because none remained statistically significant after Benjamini-Hochberg false discovery rate correction. aOR, adjusted odds ratio; CI, confidence interval; BMI, body mass index; PS, passive smoking exposure; COPD, chronic obstructive pulmonary disease; DPF, diffuse pulmonary fibrosis; ICP, irregular chest pain; COU, persistent cough; DIET, high salt or sugar intake; FDR, first-degree relative history of lung cancer; LDCT, low-dose computed tomography.

Significant variables from the overall model and nominally significant variables from the smoking-status-stratified models are summarized in Figure 2. In the overall multivariable logistic regression model, smoking status, passive smoking exposure, irregular chest pain, and persistent cough were associated with LDCT-detected pulmonary nodules ≥5 mm (Supplementary Table S2). Compared with never smokers, former smokers had lower odds of nodules ≥5 mm (aOR = 0.742, 95% CI: 0.593–0.928), as did current smokers (aOR = 0.619, 95% CI: 0.503–0.762). Passive smoking exposure was associated with higher odds of nodules ≥5 mm (aOR = 1.257, 95% CI: 1.056–1.496). In contrast, irregular chest pain (aOR = 0.709, 95% CI: 0.573–0.877) and persistent cough (aOR = 0.806, 95% CI: 0.653–0.996) were associated with lower odds of nodules ≥5 mm. Sex, age, BMI, smoking pack-years, COPD, diffuse pulmonary fibrosis, hemoptysis, hoarseness, cooking oil fume exposure, pickled or preserved food intake, high salt or sugar intake, and first-degree relative history of lung cancer were not statistically significant in the primary model. Model diagnostics are summarized in Supplementary Table S3. The overall model was statistically significant (χ^2^ = 76.950, df = 17, *p* < 0.001). Calibration was acceptable based on the Hosmer-Lemeshow goodness-of-fit test (χ^2^ = 4.375, df = 8, *p* = 0.822). The Nagelkerke *R*^2^ was 0.022. No evidence of problematic multicollinearity was observed, with VIFs ranging from 1.015 to 1.467.

### 3.3 Factors associated with pulmonary nodules ≥5 mm in participants with different smoking status

Smoking-status-stratified multivariable models are shown in Supplementary Table S4. In the never smoker subgroup, BMI (aOR = 1.031, 95% CI: 1.002–1.061), passive smoking exposure (aOR = 1.254, 95% CI: 1.019–1.545), COPD (aOR = 1.969, 95% CI: 1.046–3.708), and first-degree relative history of lung cancer (aOR = 1.276, 95% CI: 1.021–1.593) were nominally associated with higher odds of LDCT-detected pulmonary nodules ≥5 mm, whereas diffuse pulmonary fibrosis (aOR = 0.562, 95% CI: 0.337–0.935) and irregular chest pain (aOR = 0.736, 95% CI: 0.561–0.965) were nominally associated with lower odds. In former smokers, high salt or sugar intake was nominally associated with higher odds of nodules ≥5 mm (aOR = 1.661, 95% CI: 1.072–2.575). In current smokers, irregular chest pain was nominally associated with lower odds of nodules ≥5 mm (aOR = 0.561, 95% CI: 0.347–0.908). However, none of these subgroup-specific associations remained statistically significant after Benjamini-Hochberg false discovery rate correction (Supplementary Table S5); therefore, these findings should be interpreted as exploratory.

### 3.4 Sensitivity analyses

Sensitivity analyses were conducted to assess the robustness of the primary multivariable model (Supplementary Tables S6 and S7). The backward likelihood ratio sensitivity analysis generally supported the primary model, as smoking status, passive smoking exposure, irregular chest pain, and persistent cough were retained with directions of association consistent with the enter-method model. In the final backward model, the overall model remained statistically significant (χ^2^ = 69.191, df = 6, *p* < 0.001), with no evidence of poor calibration based on the Hosmer-Lemeshow test (χ^2^ = 4.381, df = 8, *p* = 0.821). Compared with never smokers, former smokers had lower odds of LDCT-detected pulmonary nodules ≥5 mm (aOR = 0.707, 95% CI: 0.577–0.865), as did current smokers (aOR = 0.593, 95% CI: 0.493–0.713). Passive smoking exposure remained associated with higher odds of nodules ≥5 mm (aOR = 1.280, 95% CI: 1.079–1.519), whereas irregular chest pain (aOR = 0.712, 95% CI: 0.577–0.877) and persistent cough (aOR = 0.808, 95% CI: 0.660–0.991) remained associated with lower odds. BMI was also retained in the final backward model, but it did not reach statistical significance (aOR = 1.021, 95% CI: 0.998–1.045).

Sequential adjustment models further supported the robustness of the inverse association between smoking status and LDCT-detected pulmonary nodules ≥5 mm. The lower odds observed among former and current smokers compared with never smokers persisted after adjustment for age and sex and remained similar after further adjustment for BMI, smoking pack-years, passive smoking exposure, and the full set of covariates (Supplementary Table S7). In the unadjusted smoking-status model, former smokers (OR = 0.700, 95% CI: 0.573–0.853) and current smokers (OR = 0.582, 95% CI: 0.486–0.696) had lower odds of nodules ≥5 mm than never smokers. After adjustment for sex and age, the associations were attenuated but remained statistically significant for both former smokers (aOR = 0.745, 95% CI: 0.599–0.928) and current smokers (aOR = 0.616, 95% CI: 0.505–0.751). Further adjustment for BMI, smoking pack-years, and passive smoking exposure produced similar estimates. In the fully adjusted model, the associations remained statistically significant for former smokers (aOR = 0.742, 95% CI: 0.593–0.928) and current smokers (aOR = 0.619, 95% CI: 0.503–0.762). These findings suggest that the lower odds observed among former and current smokers were not fully explained by differences in age and sex distribution.

## 4 Discussion

Using a large community screening cohort in Zhuhai, China, this study examined LDCT detected pulmonary nodules ≥5 mm among individuals at high risk for lung cancer and identified risk patterns across smoking groups. Three main findings emerged. First, smoking-related variables remained associated with pulmonary nodule detection, but the direction differed between active and passive smoking. Former and current smokers had lower adjusted odds of LDCT-detected pulmonary nodules ≥5 mm than never smokers, whereas passive smoking exposure was positively associated with detection. Second, factors unrelated to smoking also played an important role in pulmonary nodule detection. Smoking status, passive smoking exposure, irregular chest pain, and persistent cough were associated with pulmonary nodule detection in the overall model, while several factors showed nominal signals in exploratory smoking-status-stratified analyses. Third, perceived symptoms or medical history did not necessarily indicate higher pulmonary nodule detection, and some symptoms or medical history were instead associated with lower detection. These findings suggest that risk assessment in community based pulmonary nodule screening for subpopulations should go beyond smoking status, particularly by considering factors unrelated to smoking across different smoking groups.

### 4.1 Screening yield

Earlier and more precise detection of pulmonary nodules among individuals at high risk for lung cancer delivers clear clinical, survival, and economic benefits (34). In this study, 20.6% of participants were found to have at least one pulmonary nodule ≥5 mm. Compared with pulmonary nodule detection rates by LDCT in screening programs from inland regions such as Hebei (12.73%), Beijing (10.99%), Chongqing (12.91%), Hunan (5.92%), and Henan (5.87%), this detection rate was similar to findings from eastern and southern coastal regions of China, including Zhejiang (21.61%) and Guangzhou (19.63%) (35–37). These regional differences may reflect differences in cancer screening access and medical resources between coastal and inland areas, as well as variation in eligibility criteria, screening protocols, and nodule definitions across screening programs (38). Nevertheless, the detection yield suggests that the current community screening strategy in Zhuhai is applicable for identifying pulmonary nodules ≥5 mm among local individuals classified as being at high risk for lung cancer. Understanding the relationship between risk factors captured in screening questionnaires and pulmonary nodules may help further improve detection yield.

### 4.2 Active and passive smoking exposure

Smoking-related variables remained important in relation to pulmonary nodule detection, consistent with most previous studies (39, 40). In the overall model, former and current smokers had lower adjusted odds of LDCT detected pulmonary nodules ≥5 mm than never smokers, whereas passive smoking exposure was associated with higher odds of detection. Although current screening guidelines usually classify never smokers as a lower risk population (13), the crude detection rate of nodules ≥5 mm was highest among never smokers (23.3%). This finding may partly reflect substantial exposure to second hand smoke in households, workplaces, and other semi private environments. Such exposure may contribute to chronic airway irritation and inflammatory changes relevant to pulmonary nodule development. Evidence from a study conducted in Anhui reported lower lung cancer detection rates among smokers than among female nonsmokers exposed to second hand smoke. In that study, non-smoking women were also more likely to present with ground glass nodules detectable through LDCT (41). Similarly, regions with a high proportion of lung cancer cases among never smokers, such as Taiwan, suggest that smoking status alone may inadequately capture individuals with radiologically detectable abnormalities (42). This finding is biologically plausible, considering that second hand smoke contains numerous toxic compounds that may induce epithelial injury and remodeling in lung tissue, processes that can manifest radiologically as pulmonary nodules (43).

Several methodological and population-level factors may also have contributed to the higher observed detection among never smokers. The study population was not a general community sample, but a selected group of individuals classified as being at high risk for lung cancer who subsequently completed LDCT and uploaded imaging results. Within this screening pathway, never smokers may have entered the LDCT-completed cohort because of other risk indicators, such as passive smoke exposure, family history, respiratory symptoms, or prior lung-related history, which could make them different from never smokers in the general population (44–46). Residual confounding and selection mechanisms related to high-risk classification and LDCT completion may also have shaped the observed associations. Since the analysis was restricted to individuals classified as high risk and with completed LDCT, collider bias related to selection into the analytic cohort cannot be excluded. Sequential adjustment suggested that age and sex distributions did not fully account for the lower adjusted odds among former and current smokers. In addition, healthcare seeking behavior, risk perception, access to LDCT, and willingness to upload reports may have varied by smoking status and could not be fully measured in the present dataset (47, 48). These considerations highlight the need for more detailed assessment of passive smoking and other non-smoking indicators.

### 4.3 Non-smoking related factors

Beyond active and passive smoking, several non-smoking factors also appeared relevant to pulmonary nodule detection, particularly in exploratory smoking status stratified analyses. Household and dietary exposures were of particular interest because they are common in Guangdong and have been linked to respiratory and lung cancer risk in previous studies (49–51). Kitchen fumes are a major exposure in southern China, where stir-frying and deep-frying are common and fewer than 40% of households have both a separate kitchen and mechanical ventilation (52). Heating oils, fats, and certain food ingredients can release airborne pollutants that may contribute to chronic airway irritation and structural changes in lung tissue detectable on imaging (53). Previous studies have also linked cooking oil fume exposure with lung cancer risk (54). Similarly, preserved foods, salted foods, and high sugar intake have been proposed as relevant to pulmonary nodules through pathways involving nitrosamines, metabolic dysfunction, or inflammation (22, 55). Unexpectedly, cooking oil fume exposure and pickled or preserved food intake were not significant in any model, whereas high salt or sugar intake was nominally significant only among former smokers. One possible explanation is that the simplified self-reported indicators used in the screening questionnaire may not have captured exposure intensity, duration, ventilation conditions, food types, or long-term dietary patterns with sufficient precision.

Other non-smoking factors showed more visible signals in the exploratory smoking status stratified analyses, especially among never smokers. In this subgroup, BMI, COPD, and first-degree family history of lung cancer were nominally associated with higher odds of LDCT detected pulmonary nodules ≥5 mm. Family history may reflect shared genetic susceptibility or shared environmental exposures, and previous evidence has linked first-degree family history of lung cancer with increased lung cancer risk (4, 22, 56). This factor may be particularly relevant among never smokers, for whom familial risk can be underrecognized in screening frameworks that primarily emphasize smoking history (57, 58). The association between COPD and higher pulmonary nodule detection among never smokers may also reflect underlying airway injury, chronic inflammation, or structural lung changes that are more likely to be visible on LDCT (59).

However, self-reported symptoms and lung-related history showed a more complex pattern, which did not consistently indicate higher pulmonary nodule detection. Across all regression results, COPD among never smokers was the only lung-related history variable associated with higher detection. By contrast, irregular chest pain was associated with lower pulmonary nodule detection in the overall population, among never smokers, and among current smokers. Persistent cough was also associated with lower detection in the overall population, and diffuse pulmonary fibrosis was associated with lower detection among never smokers. This pattern may reflect the distinction between lung cancer risk assessment and pulmonary nodule detection. Small pulmonary nodules are often detected through imaging and may not cause noticeable symptoms, while their clinical evaluation depends heavily on radiological features such as size, morphology, location, and growth, together with patient-level malignancy risk (60). By contrast, symptoms such as cough, chest pain, and hemoptysis are broad clinical indicators used in lung cancer suspicion or screening eligibility, but they may also arise from heterogeneous non-nodular conditions, including airway irritation, infection, obstructive disease, or other benign respiratory disorders (45, 46, 61). In the present program, these symptoms and lung-related histories were included in the lung cancer high-risk scoring system as they have been linked to lung cancer risk. In this baseline screening cohort, symptom reporting may have helped identify individuals eligible for LDCT, but it did not consistently correspond to a higher probability of detecting pulmonary nodules ≥5 mm, suggesting a more complex relationship with LDCT detected pulmonary nodules ≥5 mm. Thus, association between lung cancer related risk indicators and pulmonary nodule detection requires further evaluation, particularly in community screening populations where questionnaire-based risk assessment is used before LDCT referral.

### 4.4 Implications for community lung cancer screening

#### 4.4.1 Strengthening risk assessment beyond active smoking

High detection rates among never smokers show that active-smoking-based eligibility alone may not adequately capture at-risk individuals. Tobacco-control policies, including smoke-free regulations and Framework Convention on Tobacco Control measures, have reduced active smoking prevalence. However, substantial second-hand smoke exposure to never smokers persists in private and semi-private settings in China (62–66). Such conditions limit the ability of smoking history to function as an effective risk filter. Screening programs should incorporate passive smoke exposure and prior respiratory conditions into eligibility assessment. Locally calibrated risk prediction tools combining environmental and familial variables may improve the identification of susceptible individuals in low-smoking regions (67). Periodic reassessment is also advisable, as exposure profiles and risk status may change over time.

#### 4.4.2 Improving questionnaire and referral design

Questionnaire based screening tools should be refined to better capture nonsmoking exposures and clinical indicators. Although cooking and diet related factors have been identified as risk factors for pulmonary nodules in previous studies, the exploratory findings in the present study showed a more complex pattern. This may be partly related to the design of the screening questionnaire. For household and dietary factors, simple yes or no response options may not provide sufficient information. Future questionnaires should collect more detailed information, such as exposure intensity, duration, kitchen ventilation, cooking methods, food types, and long-term dietary patterns (14, 68). Incorporating lifestyle counseling into the screening process may also help participants better understand nonsmoking exposures. In addition, community health education on nonsmoking factors may improve public awareness and help individuals recognize and report relevant symptoms and lifestyle related risks in a timely manner (69, 70).

#### 4.4.3 Testing generalizability through regional collaboration

The current screening strategy was able to identify pulmonary nodules ≥5 mm among individuals classified as being at high risk for lung cancer in Zhuhai, but its generalizability requires further evaluation. Future studies may test this screening strategy in neighboring regions and other community settings, using comparable eligibility criteria, LDCT protocols, and nodule definitions where possible. Multi-region collaborative screening studies would help determine whether similar detection yields can be achieved, whether passive smoking and other questionnaire-derived risk indicators show consistent associations with pulmonary nodule detection, and how risk assessment tools can be adapted for different population structures and exposure profiles.

### 4.5 Limitations

This study has several limitations. First, the analysis was restricted to high-risk participants who completed LDCT and uploaded LDCT findings. Although this restriction reduced outcome misclassification, it may have introduced LDCT completion-related selection bias; therefore, the findings may not be generalizable to all high-risk individuals identified through the community screening program. Second, pulmonary nodule status was determined from uploaded LDCT reports within the program platform, but detailed radiological characteristics were not systematically available, including solid versus subsolid or ground-glass nodules, solitary versus multiple nodules, nodule location, and radiological suspicion of malignancy. As a result, subtype-specific risk patterns could not be examined. Nevertheless, nodule size remains an important indicator of malignancy potential, and nodules ≥5 mm have been associated with higher malignant risk than smaller nodules, providing useful signals for community screening (56). Third, since this was a baseline cross-sectional analysis without longitudinal imaging follow-up, we could not determine whether nodules remained stable for a certain period of time or exclude transient nodules. Future studies may link baseline screening records with longitudinal LDCT follow-up to distinguish persistent from transient nodules and to evaluate changes in nodule size, morphology, and clinical significance. Fourth, several exposure variables, including passive smoking, cooking oil fume exposure, pickled or preserved food intake, and high salt or sugar intake, were self-reported screening-level indicators and may be affected by recall bias and exposure misclassification. Fifth, smoking-status subgroup analyses involved multiple comparisons. Although false discovery rate adjustment was applied, subgroup-specific findings should still be interpreted as exploratory. Lastly, we also noted that the Nagelkerke R^2^ of the primary model was relatively low (0.022), which is common in large-scale community screening studies evaluating highly heterogeneous outcomes based primarily on questionnaire-derived epidemiological risk factors (71). Since the study was based on community screening data, more direct clinical evidence related to pulmonary nodule development was not available. However, the aim of this study was not to develop a prediction model, but to examine associations between questionnaire-derived factors and LDCT-detected pulmonary nodules ≥5 mm. The current results therefore remain adequate for addressing this objective.

## 5 Conclusion

This real-world study provides valuable empirical evidence for smoking status stratified pulmonary nodule detection within community lung cancer screening in China. Pulmonary nodules ≥5 mm were detected in approximately one fifth of LDCT completed participants, with the highest detection among never smokers; passive smoking was positively associated with detection, whereas symptom and lung related history indicators showed inconsistent patterns. Community screening should enhance risk assessment beyond active smoking, refine questionnaire based nonsmoking indicators, and evaluate the generalizability of this screening protocol through multi region collaborative studies.

## Data Availability

The datasets presented in this article are not readily available because the data that support the findings of this study are not publicly available due to data use agreements with the Zhuhai Medical Insurance Bureau. Access may be granted upon reasonable request through the official government information disclosure system. Requests to access the datasets should be directed to https://ysqgk.gd.gov.cn/756194/choice.
