# Supplementary material for: Beyond smoking status: risk factors for pulmonary nodules ≥5 mm in a Chinese community screening program
Source: Front Med (Lausanne). 2026 Jul 22;13:1857233. doi: 10.3389/fmed.2026.1857233 (PMC13438382; doi:10.3389/fmed.2026.1857233)
Supplement: Supplementary file 1 [file Supplementary_file_1.docx]

Supplementary Material

For

*Beyond Smoking Status: Risk Factors for Pulmonary Nodules ≥5 mm in a Chinese Community Screening Program*

| Content | Sub-content | Page |
| --- | --- | --- |
| S1. Supplementary Tables | Supplementary Table S1. Missing data for variables included in the final model analysis | 2 |
|  | Supplementary Table S2. Multivariable logistic regression for LDCT-detected pulmonary nodules ≥5 mm in the overall cohort | 3 |
|  | Supplementary Table S3. Model diagnostics for the primary multivariable logistic regression model | 4 |
|  | Supplementary Table S4. Smoking-status-stratified multivariable logistic regression models for LDCT-detected pulmonary nodules ≥5 mm | 5 |
|  | Supplementary Table S5. Benjamini–Hochberg false discovery rate assessment for smoking-status-stratified analyses | 7 |
|  | Supplementary Table S6. Backward likelihood ratio sensitivity analysis for LDCT-detected pulmonary nodules ≥5 mm | 8 |
|  | Supplementary Table S7. Sequential adjustment models for the association between smoking status and LDCT-detected pulmonary nodules ≥5 mm | 9 |
| S2. STROBE Statement checklist for cross-sectional studies | | 11-13 |
| S3. Zhuhai Supplementary Medical Insurance Cancer Screening Questionnaire (Chinese–English Bilingual Version) | | 14-25 |

# 1 Supplementary Tables

**Supplementary Table S1. Missing data for variables included in the final model analysis**

|  | | PN | SEX | AGE | BMI | SMK | SPY | PS | COPD | DPF | ICP | HEM | COU | HOA | COOK | FOOD | DIET | FDR |
| --- | --- | --- | --- | --- | --- | --- | --- | --- | --- | --- | --- | --- | --- | --- | --- | --- | --- | --- |
| N | Valid | 5414 | 5414 | 5414 | 5414 | 5414 | 5414 | 5414 | 5414 | 5414 | 5414 | 5414 | 5414 | 5414 | 5414 | 5414 | 5414 | 5414 |
|  | Missing | 0 | 0 | 0 | 0 | 0 | 0 | 0 | 0 | 0 | 0 | 0 | 0 | 0 | 0 | 0 | 0 | 0 |

**Note.** This table summarizes missing data for the outcome and all variables included in the final model analysis among LDCT-completed high-risk participants. All variables had complete coded values in the final analytic sample (N = 5,414). PN, pulmonary nodules ≥5 mm; SEX, sex assigned at birth; AGE, age; BMI, body mass index; SMK, smoking status; SPY, smoking pack-years; PS, passive smoking exposure; COPD, chronic obstructive pulmonary disease; DPF, diffuse pulmonary fibrosis; ICP, irregular chest pain; HEM, hemoptysis; COU, persistent cough; HOA, hoarseness; COOK, cooking oil fume exposure; FOOD, pickled or preserved food intake; DIET, high salt or sugar intake; FDR, first-degree relative history of lung cancer.

**Supplementary Table S2. Multivariable logistic regression for LDCT-detected pulmonary nodules ≥5 mm in the overall cohort**

| **Variable** | **aOR** | **95% CI** | **p value** |
| --- | --- | --- | --- |
| SEX, female vs male | 1.121 | 0.956–1.316 | 0.160 |
| AGE, per year | 1.000 | 0.993–1.006 | 0.918 |
| BMI, per kg/m² | 1.022 | 0.999–1.046 | 0.064 |
| SMK, overall | — | — | <0.001*** |
| Former smoker vs never smoker | 0.742 | 0.593–0.928 | 0.009** |
| Current smoker vs never smoker | 0.619 | 0.503–0.762 | <0.001*** |
| SPY, per pack-year | 1.000 | 0.999–1.001 | 0.711 |
| PS, yes vs no | 1.257 | 1.056–1.496 | 0.010* |
| COPD, yes vs no | 1.068 | 0.684–1.668 | 0.773 |
| DPF, yes vs no | 0.753 | 0.509–1.114 | 0.155 |
| ICP, yes vs no | 0.709 | 0.573–0.877 | 0.002** |
| HEM, yes vs no | 0.956 | 0.622–1.469 | 0.838 |
| COU, yes vs no | 0.806 | 0.653–0.996 | 0.045* |
| HOA, yes vs no | 0.998 | 0.770–1.295 | 0.990 |
| COOK, yes vs no | 0.920 | 0.726–1.165 | 0.488 |
| FOOD, yes vs no | 1.166 | 0.899–1.512 | 0.248 |
| DIET, yes vs no | 1.052 | 0.874–1.267 | 0.590 |
| FDR, yes vs no | 1.110 | 0.932–1.323 | 0.243 |

**Note.** Adjusted odds ratios were estimated using multivariable logistic regression with the enter method. The dependent variable was LDCT-detected pulmonary nodules ≥5 mm. Never smokers were used as the reference group for smoking status. ORs for age, BMI, and smoking pack-years correspond to one-unit increases. *p <0.05; **p <0.01; ***p <0.001. aOR, adjusted odds ratio; CI, confidence interval; AGE, age; BMI, body mass index; SMK, smoking status; SPY, smoking pack-years; PS, passive smoking exposure; COPD, chronic obstructive pulmonary disease; DPF, diffuse pulmonary fibrosis; ICP, irregular chest pain; HEM, hemoptysis; COU, persistent cough; HOA, hoarseness; COOK, cooking oil fume exposure; FOOD, pickled or preserved food intake; DIET, high salt or sugar intake; FDR, first-degree relative history of lung cancer.

**Supplementary Table S3. Model diagnostics for the primary multivariable logistic regression model**

| **Diagnostic item** | **Result** |
| --- | --- |
| Included cases | 5,414 |
| Missing cases | 0 |
| Outcome events | 1,117 |
| Non-events | 4,297 |
| Predictor parameters | 17 |
| Events per predictor parameter | 65.7 |
| Omnibus test of model coefficients | χ² = 76.950, df = 17, p <0.001 |
| −2 Log likelihood | 5,434.890 |
| Cox & Snell R² | 0.014 |
| Nagelkerke R² | 0.022 |
| Hosmer–Lemeshow goodness-of-fit test | χ² = 4.375, df = 8, p = 0.822 |
| Tolerance range | 0.682–0.985 |
| VIF range | 1.015–1.467 |

**Note.** The primary model was fitted using multivariable logistic regression with the enter method. The dependent variable was LDCT-detected pulmonary nodules ≥5 mm. Events per predictor parameter were calculated as 1,117 events divided by 17 predictor parameters. Variance inflation factors (VIFs) were calculated using the same predictor set as the primary model, with smoking status represented by former-smoker and current-smoker dummy variables and never smokers as the reference group. LDCT, low-dose computed tomography; VIF, variance inflation factor.

**Supplementary Table S4. Smoking-status-stratified multivariable logistic regression models for LDCT-detected pulmonary nodules ≥5 mm**

| **Variable** | **Never smokers (n = 3,447; events = 802), aOR (95% CI)** | **p value** | **Former smokers (n = 800; events = 140), aOR (95% CI)** | **p value** | **Current smokers (n = 1,167; events = 175), aOR (95% CI)** | **p value** |
| --- | --- | --- | --- | --- | --- | --- |
| SEX, female vs male | 1.133 (0.942–1.362) | 0.185 | 1.362 (0.796–2.330) | 0.260 | 1.051 (0.679–1.627) | 0.822 |
| AGE, per year | 0.998 (0.990–1.006) | 0.596 | 1.012 (0.993–1.031) | 0.234 | 0.993 (0.977–1.010) | 0.419 |
| BMI, per kg/m² | 1.031 (1.002–1.061) | 0.037* | 0.996 (0.934–1.063) | 0.909 | 1.016 (0.964–1.071) | 0.549 |
| SPY, per pack-year | Not included | — | 1.009 (0.998–1.020) | 0.109 | 1.000 (0.998–1.001) | 0.687 |
| PS, yes vs no | 1.254 (1.019–1.545) | 0.033* | 1.484 (0.928–2.372) | 0.099 | 1.148 (0.738–1.785) | 0.540 |
| COPD, yes vs no | 1.969 (1.046–3.708) | 0.036* | 0.711 (0.279–1.808) | 0.474 | 0.416 (0.144–1.203) | 0.105 |
| DPF, yes vs no | 0.562 (0.337–0.935) | 0.027* | 0.864 (0.281–2.657) | 0.798 | 1.296 (0.603–2.788) | 0.507 |
| ICP, yes vs no | 0.736 (0.561–0.965) | 0.027* | 0.738 (0.431–1.262) | 0.267 | 0.561 (0.347–0.908) | 0.019* |
| HEM, yes vs no | 1.209 (0.721–2.028) | 0.472 | 0.424 (0.095–1.892) | 0.261 | 0.868 (0.317–2.379) | 0.783 |
| COU, yes vs no | 0.809 (0.616–1.064) | 0.130 | 0.542 (0.293–1.003) | 0.051 | 0.937 (0.625–1.405) | 0.753 |
| HOA, yes vs no | 1.060 (0.771–1.458) | 0.721 | 1.501 (0.796–2.832) | 0.209 | 0.599 (0.299–1.200) | 0.148 |
| COOK, yes vs no | 0.986 (0.747–1.300) | 0.919 | 0.774 (0.388–1.545) | 0.468 | 0.691 (0.380–1.257) | 0.226 |
| FOOD, yes vs no | 1.175 (0.877–1.574) | 0.280 | 2.012 (0.686–5.899) | 0.203 | 0.807 (0.403–1.615) | 0.545 |
| DIET, yes vs no | 0.894 (0.698–1.144) | 0.372 | 1.661 (1.072–2.575) | 0.023* | 1.170 (0.798–1.714) | 0.421 |
| FDR, yes vs no | 1.276 (1.021–1.593) | 0.032* | 0.751 (0.459–1.230) | 0.255 | 0.964 (0.667–1.394) | 0.845 |

**Note.** Models were fitted separately within each smoking-status subgroup using multivariable logistic regression with the enter method. The dependent variable was LDCT-detected pulmonary nodules ≥5 mm. Smoking status was not included in subgroup-specific models. Smoking pack-years were not included in the never-smoker model because they were uniformly coded as 0 after logical consistency checks. ORs for age, BMI, and smoking pack-years correspond to one-unit increases. Asterisks indicate nominal statistical significance before multiple-comparison correction: *p <0.05; **p <0.01; ***p <0.001. Subgroup-specific findings should be interpreted as exploratory. LDCT, low-dose computed tomography; aOR, adjusted odds ratio; CI, confidence interval; AGE, age; BMI, body mass index; SPY, smoking pack-years; PS, passive smoking exposure; COPD, chronic obstructive pulmonary disease; DPF, diffuse pulmonary fibrosis; ICP, irregular chest pain; HEM, hemoptysis; COU, persistent cough; HOA, hoarseness; COOK, cooking oil fume exposure; FOOD, pickled or preserved food intake; DIET, high salt or sugar intake; FDR, first-degree relative history of lung cancer.

**Supplementary Table S5. Benjamini–Hochberg false discovery rate assessment for smoking-status-stratified analyses**

| **Smoking group** | **Number of tested predictors** | **Smallest raw p value** | **Smallest BH-adjusted p value** | **Significant after BH-FDR correction?** |
| --- | --- | --- | --- | --- |
| Never smokers | 14 | 0.027 | 0.086 | No |
| Former smokers | 15 | 0.023 | 0.345 | No |
| Current smokers | 15 | 0.019 | 0.285 | No |

**Note.** Benjamini–Hochberg false discovery rate (BH-FDR) correction was applied separately within each smoking-status subgroup. The never-smoker model included 14 tested predictors because smoking pack-years were not included in this subgroup. The former-smoker and current-smoker models each included 15 tested predictors. The table reports the smallest raw p value and the corresponding smallest BH-adjusted p value within each subgroup. No subgroup-specific association remained statistically significant after BH-FDR correction at q <0.05. Therefore, findings from smoking-status-stratified analyses were interpreted as exploratory. BH-FDR, Benjamini–Hochberg false discovery rate.

**Supplementary Table S6. Backward likelihood ratio sensitivity analysis for LDCT-detected pulmonary nodules ≥5 mm**

| **Variable retained in the final model** | **aOR** | **95% CI** | **p value** |
| --- | --- | --- | --- |
| BMI, per kg/m² | 1.021 | 0.998–1.045 | 0.076 |
| SMK, overall | — | — | <0.001*** |
| Former smoker vs never smoker | 0.707 | 0.577–0.865 | 0.001** |
| Current smoker vs never smoker | 0.593 | 0.493–0.713 | <0.001*** |
| PS, yes vs no | 1.280 | 1.079–1.519 | 0.005** |
| ICP, yes vs no | 0.712 | 0.577–0.877 | 0.001** |
| COU, yes vs no | 0.808 | 0.660–0.991 | 0.040* |

**Additional model diagnostics.** Included cases = 5,414; missing cases = 0; −2 Log likelihood = 5,442.650; Cox & Snell R² = 0.013; Nagelkerke R² = 0.020; Omnibus model test χ² = 69.191, df = 6, p <0.001; Hosmer–Lemeshow test χ² = 4.381, df = 8, p = 0.821.

**Note.** Backward likelihood ratio selection was performed as a sensitivity analysis. The dependent variable was LDCT-detected pulmonary nodules ≥5 mm. The initial model included the same candidate variables as the primary enter-method model: sex, age, BMI, smoking status, smoking pack-years, passive smoking exposure, COPD, diffuse pulmonary fibrosis, irregular chest pain, hemoptysis, persistent cough, hoarseness, cooking oil fume exposure, pickled or preserved food intake, high salt or sugar intake, and first-degree relative history of lung cancer. Variables removed during backward selection were hoarseness, age, hemoptysis, COPD, smoking pack-years, high salt or sugar intake, cooking oil fume exposure, pickled or preserved food intake, first-degree relative history of lung cancer, diffuse pulmonary fibrosis, and sex. *p <0.05; **p <0.01; ***p <0.001. LDCT, low-dose computed tomography; aOR, adjusted odds ratio; CI, confidence interval; BMI, body mass index; SMK, smoking status; PS, passive smoking exposure; ICP, irregular chest pain; COU, persistent cough.

**Supplementary Table S7. Sequential adjustment models for the association between smoking status and LDCT-detected pulmonary nodules ≥5 mm**

| **Model** | **Covariates adjusted** | **Former smoker vs never smoker, OR (95% CI)** | **p value** | **Current smoker vs never smoker, OR (95% CI)** | **p value** | **Nagelkerke R²** |
| --- | --- | --- | --- | --- | --- | --- |
| Model 1 | Smoking status only | 0.700 (0.573–0.853) | <0.001*** | 0.582 (0.486–0.696) | <0.001*** | 0.013 |
| Model 2 | Model 1 + sex + age | 0.745 (0.599–0.928) | 0.009** | 0.616 (0.505–0.751) | <0.001*** | 0.013 |
| Model 3 | Model 2 + BMI + smoking pack-years + passive smoking exposure | 0.732 (0.587–0.913) | 0.006** | 0.599 (0.490–0.733) | <0.001*** | 0.016 |
| Model 4 | Model 3 + COPD + diffuse pulmonary fibrosis + irregular chest pain + hemoptysis + persistent cough + hoarseness + cooking oil fume exposure + pickled or preserved food intake + high salt or sugar intake + first-degree relative history of lung cancer | 0.742 (0.593–0.928) | 0.009** | 0.619 (0.503–0.762) | <0.001*** | 0.022 |

**Note.** Sequential logistic regression models were fitted to examine whether the association between smoking status and LDCT-detected pulmonary nodules ≥5 mm was explained by age and sex or by additional covariates. The dependent variable was LDCT-detected pulmonary nodules ≥5 mm. Never smokers were used as the reference group for smoking status. Model 4 corresponds to the primary fully adjusted enter-method model. *p <0.05; **p <0.01; ***p <0.001. LDCT, low-dose computed tomography; aOR, adjusted odds ratio; CI, confidence interval; BMI, body mass index; COPD, chronic obstructive pulmonary disease.

# 2 STROBE Statement checklist for cross-sectional studies

|  | **Item No** | **Recommendation** | **Page No** |
| --- | --- | --- | --- |
| **Title and abstract** | 1 | (*a*) Indicate the study’s design with a commonly used term in the title or the abstract | 1 |
|  |  | (*b*) Provide in the abstract an informative and balanced summary of what was done and what was found | 1, 2 |
| **Introduction** | | | |
| Background/rationale | 2 | Explain the scientific background and rationale for the investigation being reported | 2, 3 |
| Objectives | 3 | State specific objectives, including any prespecified hypotheses | 3 |
| **Methods** | | | |
| Study design | 4 | Present key elements of study design early in the paper | 3 |
| Setting | 5 | Describe the setting, locations, and relevant dates, including periods of recruitment, exposure, follow-up, and data collection | 3 |
| Participants | 6 | (*a*) Give the eligibility criteria, and the sources and methods of selection of participants | 3 |
| Variables | 7 | Clearly define all outcomes, exposures, predictors, potential confounders, and effect modifiers. Give diagnostic criteria, if applicable | 3, 4 |
| Data sources/ measurement | 8* | For each variable of interest, give sources of data and details of methods of assessment (measurement). Describe comparability of assessment methods if there is more than one group | 3, 4 |
| Bias | 9 | Describe any efforts to address potential sources of bias | 4 |
| Study size | 10 | Explain how the study size was arrived at | 3, 4 |
| Quantitative variables | 11 | Explain how quantitative variables were handled in the analyses. If applicable, describe which groupings were chosen and why | 4 |
| Statistical methods | 12 | (*a*) Describe all statistical methods, including those used to control for confounding | 4, 5 |
|  |  | (*b*) Describe any methods used to examine subgroups and interactions | 5 |
|  |  | (*c*) Explain how missing data were addressed | 4 |
|  |  | (*d*) If applicable, describe analytical methods taking account of sampling strategy | 4 |
|  |  | (*e*) Describe any sensitivity analyses | 5 |
| **Results** | | | |
| Participants | 13* | (a) Report numbers of individuals at each stage of study—eg numbers potentially eligible, examined for eligibility, confirmed eligible, included in the study, completing follow-up, and analysed | 5, 6 |
|  |  | (b) Give reasons for non-participation at each stage | NA |
|  |  | (c) Consider use of a flow diagram | *Figure 1* |
| Descriptive data | 14* | (a) Give characteristics of study participants (eg demographic, clinical, social) and information on exposures and potential confounders | 5, 6, Table 3 |
|  |  | (b) Indicate number of participants with missing data for each variable of interest | 4, Supplementary Table S1 |
| Outcome data | 15* | Report numbers of outcome events or summary measures | 5 |
| Main results | 16 | (*a*) Give unadjusted estimates and, if applicable, confounder-adjusted estimates and their precision (eg, 95% confidence interval). Make clear which confounders were adjusted for and why they were included | 6 |
|  |  | (*b*) Report category boundaries when continuous variables were categorized | 6 |
|  |  | (*c*) If relevant, consider translating estimates of relative risk into absolute risk for a meaningful time period | NA |
| Other analyses | 17 | Report other analyses done—eg analyses of subgroups and interactions, and sensitivity analyses | 6, 7 |
| **Discussion** | | | |
| Key results | 18 | Summarise key results with reference to study objectives | 7 |
| Limitations | 19 | Discuss limitations of the study, taking into account sources of potential bias or imprecision. Discuss both direction and magnitude of any potential bias | 10 |
| Interpretation | 20 | Give a cautious overall interpretation of results considering objectives, limitations, multiplicity of analyses, results from similar studies, and other relevant evidence | 7-10 |
| Generalisability | 21 | Discuss the generalisability (external validity) of the study results | 10 |
| **Other information** | | | |
| Funding | 22 | Give the source of funding and the role of the funders for the present study and, if applicable, for the original study on which the present article is based | 11 |

*Give information separately for exposed and unexposed groups.

**Note:** An Explanation and Elaboration article discusses each checklist item and gives methodological background and published examples of transparent reporting. The STROBE checklist is best used in conjunction with this article (freely available on the Web sites of PLoS Medicine at http://www.plosmedicine.org/, Annals of Internal Medicine at http://www.annals.org/, and Epidemiology at http://www.epidem.com/). Information on the STROBE Initiative is available at www.strobe-statement.org.

# 3 Zhuhai Supplementary Medical Insurance Cancer Screening Questionnaire (Chinese–English Bilingual Version)

**Author note**:

1. This questionnaire was originally designed as a universal screening tool for 10 types of cancer. Therefore, many items relate to cancers of other systems.

2. The original questionnaire was developed in simplified Chinese and was available in online formats. The online version could be accessed via the official WeChat service account of Zhuhai Supplementary Medical Insurance (link: <https://gdwx2.e-chinalife.com/dawj/love-health/love-health/gdwx/health/mobile.html#/asHome?key=asHome> ; accessible only via the WeChat app).

3. The English version provided in this supplementary file was translated by the research team and is intended for peer review reference.

**用户信息/User Information**

| **Chinese version** | **English version** |
| --- | --- |
| 1、填写人姓名　联系电话： | 1. Name Phone number: |
| 2、证件类型：□ 身份证 □ 港澳通行证 □ 护照　证件号码： | 2. ID type: □ Mainland ID □ Mainland Travel Permit (HK/Macao) □ Passport ID No.: |
| 3、所在区域：□ 香洲区 □ 金湾区 □ 斗门区 □ 粤澳深度合作区 □ 高新区 | 3. Residential area: □ Xiangzhou □ Jinwan □ Doumen □ Guangdong–Macao In-Depth Cooperation Zone □ High-tech Zone |

**A、基础信息/A. Basic Information**

| **Chinese version** | **English version** |
| --- | --- |
| A1、请问您的身高是？请填写　　　厘米（cm） | A1. What is your height? ______ cm |
| A2、请问您的体重是？请填写　　　公斤（kg） | A2. What is your weight? ______ kg |

**B、生活指标/B. Lifestyle Indicators**

| **Chinese version** | **English version** |
| --- | --- |
| **B1、饮食习惯/ B1. Diet habits** | |
| B1.1、您是否经常进食过热食（饮）品导致食管烫伤出现不适感？1．从不 2．有时 3．经常 | B1.1. Do you experience occasional oesophageal burns from very hot foods or beverages?1. Never 2. Sometimes 3. Often |
| B1.2、您经常食用腌晒食品（咸鱼、酸菜、泡菜、咸菜等）吗？1．从不 2．有时 3．经常 | B1.2. Do you frequently consume pickled/preserved foods (e.g., salted fish, pickled vegetables, kimchi)?1. Never 2. Sometimes 3. Often |
| B1.3、您平时的饮食口味习惯：1．重盐或重糖 2．适中 3．清淡 | B1.3. What is your usual taste preference?1. High salt/sugar intake 2. Moderate 3. Light |
| B1.4、您在家常用的烹饪方式？（多选）1．油炸 2．蒸煮 3．炒 4．煎 5．焖 6．烘烤 7．凉拌 | B1.4. Which home cooking methods do you usually use? (multiple choice)1. Deep-frying 2. Steaming/Boiling 3. Stir-frying 4. Pan-frying 5. Braising/Stewing 6. Baking/Roasting 7. Cold/No-cook |
| B1.5、您日常生活中食物的荤素搭配习惯1．荤大于素 2．荤素均衡 3．素大于荤 | B1.5. Your usual balance of meat and vegetables1. More meat than vegetables 2. Balanced 3. More vegetables than meat |
| **B2、生活环境、方式和习惯/ B2. Living environment, lifestyle and habits** | |
| B2.1、您是否吸烟？1．否，从不吸（跳转至 B2.4） 2．是，目前仍在吸 3．以前吸，目前已戒烟 | B2.1. What is your smoking status?1. Never smoker (skip to B2.4) 2. Current smoker 3. Former smoker |
| B2.2、如果您仍在吸烟或曾吸烟，平均每天吸烟　　　支？ | B2.2. If you are a current/former smoker, average cigarettes per day: ______ |
| B2.3、如果您仍在吸烟或曾吸烟，吸烟年数为　　　年； | B2.3. If you are a current/former smoker, years of smoking: ______ years |
| B2.4、若您不是吸烟者，您是否长期处于被动吸烟环境（身边长期有人吸烟）？1．是 2．否（跳转至 B2.6） | B2.4. If you are not a smoker, do you have long-term passive smoking exposure?1. Yes 2. No (skip to B2.6) |
| B2.5、若您不是吸烟者，您在以下哪种环境中被动吸烟？（多选）1．家中 2．工作环境中 3．公共场所 4．娱乐场所 5．其他 | B2.5. If you are not a smoker, where does passive smoking occur? (multiple choice)1. At home 2. Workplace 3. Public places 4. Entertainment venues 5. Other |
| B2.6、您是否经常饮酒？1．否，从不饮（跳转至 B2.7） 2．偶尔饮酒（跳转至 B2.7） 3．是，目前仍经常饮 4．以前是，目前已戒酒（跳转至 B2.7） | B2.6. Do you have regular alcohol consumption?1. No, never (skip to B2.7) 2. Occasionally (skip to B2.7) 3. Yes, currently regular 4. Previously regular, now quit (skip to B2.7) |
| B2.6.1、如果您仍在饮酒，您最常饮酒的种类是：1．啤酒 2．葡萄酒 3．38％低度白酒 4．53％高度白酒 5．其他类型酒类 | B2.6.1. If still drinking, your main type of alcohol is:1. Beer 2. Wine 3. 38% low-proof baijiu 4. 53% high-proof baijiu 5. Other |
| B2.6.2、如果您仍在饮酒，平均每天饮酒为　　　两（1 两＝50g）； | B2.6.2. If still drinking, average daily alcohol intake: ______ liang (1 liang = 50 g) |
| B2.6.3、如果您仍在饮酒，饮酒年数为　　　年（不足一年按一年计）； | B2.6.3. If still drinking, years of drinking: ______ years (less than 1 year counted as 1) |
| B2.7、您是否经常参加体育锻炼（一般指平均每周 3 次以上，每次超过 30 分钟）1．是 2．否（跳转至 C1 题） | B2.7. Do you engage in weekly regular exercise (generally ≥3 times/week, ≥30 minutes each)?1. Yes 2. No (skip to C1) |
| B2.7.1、若您经常参加体育锻炼，您经常参加的运动类型是？（多选）1．骑自行车 2．跑步或快走 3．跳绳 4．游泳 5．球类运动 6．器械运动 7．广场舞、太极拳、广播操 8．平板支撑、俯卧撑、仰卧起坐 | B2.7.1. If yes, which types of exercise do you do? (multiple choice)1. Cycling 2. Running/Brisk walking 3. Skipping rope 4. Swimming 5. Ball sports 6. Resistance training 7. Group dance/Tai-chi/Calisthenics 8. Plank/Push-ups/Sit-ups |
| B2.8、您是否经常接触放射线或从事频繁受到电离辐射的工作？（如地铁安检工作、食品辐照车间工作、医学影像学相关工作等）1．是 2．否 | B2.8. Do you have frequent occupational exposure to ionising radiation? (e.g., metro security screening, food irradiation, medical imaging)1. Yes 2. No |

**C、临床症状/C. Clinical Symptoms**

| **Chinese version** | **English version** |
| --- | --- |
| C1、您近期（一个月内）是否出现以下症状？（多选） 1．健康，无相关症状；2．持续发热；3．严重出汗；4．长期大便形状改变；5．胸闷；6．腋窝淋巴结肿胀；7．痰中带血；8．长期咳嗽；9．咯血；10．反复出现大便黑色；11．反复头晕头痛；12．乏力；13．消瘦（体重骤减5千克以上）；14．声音嘶哑；15．吞咽困难；16．有头颈部肿块；17．头颈部淋巴结肿胀；18．食欲减退；19．反复胃痛，经治疗效果不佳；20．心悸；21．双下肢伴水肿；22．反复出现反酸伴有食道疼痛症状；23．长期鼻音重，长期鼻塞（近三个月）；24．甲状腺肿大；25．偏头痛／后枕痛；26．耳鸣及听力下降；27．回吸性涕血；28．胸部不规则隐痛、钝痛一个月以上；29．乳腺肿块；30．乳头、乳晕异常；31．乳头溢液；32．乳腺皮肤改变；33．肝区持续性疼痛；34．黄疸；35．腹水；36．右上腹部包块；37．恶心呕吐；38．腹胀；39．肝肿大；40．便秘；41．腹泻；42．排便困难；43．阴道接触性出血；44．经期延长或经量增多；45．阴道异常排液或出血；46．尿频、尿急、尿痛；47．夜尿增多；48．尿不尽、尿线细、血尿；49．射精缺乏或血精；50．同房时出血；51．雄性激素分泌旺盛 | C1. In the past month, have you experienced any of the following symptoms? (multiple choice) 1. Healthy, no relevant symptoms; 2. Persistent fever; 3. Severe sweating; 4. Long-term change in stool shape; 5. Chest tightness; 6. Axillary lymph node swelling; 7. Blood-streaked sputum; 8. Persistent cough; 9. Haemoptysis; 10. Recurrent black stools; 11. Recurrent dizziness or headache; 12. Fatigue; 13. Unexplained weight loss (≥5 kg); 14. Hoarseness; 15. Dysphagia; 16. Head or neck mass; 17. Cervical lymph node swelling; 18. Loss of appetite; 19. Recurrent gastric pain with poor treatment response; 20. Palpitations; 21. Bilateral lower limb edema; 22. Recurrent acid reflux with esophageal pain; 23. Persistent nasal obstruction (≥3 months); 24. Thyroid enlargement; 25. Migraine/occipital pain; 26. Tinnitus and hearing loss; 27. Blood-tinged nasal discharge; 28. Irregular chest pain >1 month; 29. Breast lump; 30. Nipple/areola abnormality; 31. Nipple discharge; 32. Breast skin changes; 33. Persistent liver pain; 34. Jaundice; 35. Ascites; 36. Right upper abdominal mass; 37. Nausea and vomiting; 38. Abdominal distension; 39. Hepatomegaly; 40. Constipation; 41. Diarrhea; 42. Difficulty in defecation; 43. Postcoital bleeding; 44. Prolonged or heavy menstruation; 45. Abnormal vaginal discharge or bleeding; 46. Urinary frequency, urgency, or dysuria; 47. Increased nocturia; 48. Incomplete voiding, weak urinary stream, or hematuria; 49. Lack of ejaculation or hematospermia; 50. Bleeding during intercourse; 51. Signs of hyperandrogenism |
| C2、【呼吸系统】您是否曾经确诊以下疾病？（经正规医疗机构诊断）（多选） 1．否；2．肺结核；3．慢性阻塞性肺病；4．肺气肿；5．支气管哮喘；6．慢性支气管炎；7．支气管扩张；8．慢性肺炎；9．弥漫性肺纤维化病；10．肺结节；11．鼻咽黏膜上皮鳞状化生；12．鼻咽黏膜上皮异型增生 | C2. [Respiratory system] Have you ever been diagnosed with any of the following by a certified medical institution? (multiple choice) 1. No; 2. Tuberculosis; 3. Chronic obstructive pulmonary disease (COPD); 4. Emphysema; 5. Bronchial asthma; 6. Chronic bronchitis; 7. Bronchiectasis; 8. Chronic pneumonia; 9. Diffuse pulmonary fibrosis; 10. Pulmonary nodules; 11. Squamous metaplasia of nasopharyngeal epithelium; 12. Dysplasia of nasopharyngeal epithelium |
| C3、【上消化系统】您是否曾经确诊以下疾病？（多选） 1．否；2．反流性食管炎；3．食管溃疡；4．贲门失弛缓症；5．食管静脉曲张；6．食管息肉；7．食管不典型增生；8．肥厚性胃炎；9．恶性贫血；10．萎缩性胃炎；11．胃溃疡；12．胃部不典型增生；13．十二指肠溃疡；14．胃息肉病；15．手术后残胃；16．胃粘膜异型增生；17．肠上皮化生 | C3. [Upper digestive system] Have you ever been diagnosed with any of the following by a certified medical institution? (multiple choice) 1. No; 2. Reflux esophagitis; 3. Esophageal ulcer; 4. Achalasia; 5. Esophageal varices; 6. Esophageal polyps; 7. Esophageal atypical hyperplasia; 8. Hypertrophic gastritis; 9. Pernicious anemia; 10. Atrophic gastritis; 11. Gastric ulcer; 12. Gastric atypical hyperplasia; 13. Duodenal ulcer; 14. Gastric polyposis; 15. Post-gastrectomy remnant stomach; 16. Gastric mucosal dysplasia; 17. Intestinal metaplasia |
| C4、【下消化系统】您是否曾经确诊以下疾病？（多选） 1．否；2．肠息肉，且3年内未复查；3．慢性结（直）肠炎；4．慢性便秘（近2年来便秘每年在2个月以上）；5．肠易激综合症；6．黏液血便；7．慢性腹泻（近2年来累计持续3个月以上，每次发作持续1周以上）；8．慢性阑尾炎或阑尾切除史；9．慢性胆道疾病史或胆囊切除史；10．肠道憩室伴炎症 | C4. [Lower digestive system] Have you ever been diagnosed with any of the following by a certified medical institution? (multiple choice) 1. No; 2. Intestinal polyps without follow-up in past 3 years; 3. Chronic colitis/rectocolitis; 4. Chronic constipation (≥2 months/year over past 2 years); 5. Irritable bowel syndrome; 6. Mucus and bloody stool; 7. Chronic diarrhea (>3 months cumulative in past 2 years, each episode >1 week); 8. Chronic appendicitis or history of appendectomy; 9. Chronic biliary disease or history of cholecystectomy; 10. Intestinal diverticulitis |
| C5、【甲状腺】您是否曾经确诊以下疾病？（多选） 1．否；2．甲状腺功能亢进症；3．亚临床甲状腺功能减退症；4．甲状腺炎；5．甲状腺瘤；6．甲状腺肿大；7．甲状腺增生；8．毒性弥漫性甲状腺肿；9．甲状腺囊肿；10．甲状腺结节；11．家族性多发性息肉病 | C5. [Thyroid] Have you ever been diagnosed with any of the following by a certified medical institution? (multiple choice) 1. No; 2. Hyperthyroidism; 3. Subclinical hypothyroidism; 4. Thyroiditis; 5. Thyroid adenoma; 6. Goiter; 7. Thyroid hyperplasia; 8. Toxic diffuse goiter; 9. Thyroid cyst; 10. Thyroid nodule; 11. Familial multiple polyposis |
| C6、【肝脏】您是否曾经确诊以下疾病？（多选） 1．否；2．病毒性肝炎或肝硬化（含乙肝、丙肝等）；3．酒精性肝炎；4．非酒精性脂肪性肝病；5．肝淤血；6．慢性肝病；7．肝血管瘤；8．肝脏腺瘤样增生；9．肝结节；10．血吸虫病等肝脏寄生虫病 | C6. [Liver] Have you ever been diagnosed with any of the following by a certified medical institution? (multiple choice) 1. No; 2. Viral hepatitis or cirrhosis (including HBV, HCV, etc.); 3. Alcoholic hepatitis; 4. Non-alcoholic fatty liver disease; 5. Hepatic congestion; 6. Chronic liver disease; 7. Hepatic hemangioma; 8. Hepatic adenomatous hyperplasia; 9. Liver nodule; 10. Schistosomiasis or other hepatic parasitic diseases |
| C7、【泌尿与生殖系统】您是否曾经确诊以下疾病？（多选） 1．否；2．尿道炎；3．尿道结石；4．慢性肾炎；5．肾结石；6．膀胱炎；7．肾盂积水；8．输尿管积水；9．盆腔炎；10．附件炎；11．宫颈炎；12．宫颈肌瘤；13．宫颈鳞状上皮内病变；14．宫颈腺上皮内瘤变；15．前列腺炎；16．前列腺结节；17．精索静脉曲张；18．前列腺异常钙化；19．前列腺萎缩 | C7. [Urinary and Reproductive System] Have you ever been diagnosed with any of the following by a certified medical institution? (multiple choice) 1. No; 2. Urethritis; 3. Urethral calculus; 4. Chronic nephritis; 5. Renal calculus; 6. Cystitis; 7. Hydronephrosis; 8. Ureteral hydronephrosis; 9. Pelvic inflammatory disease; 10. Adnexitis; 11. Cervicitis; 12. Cervical myoma; 13. Cervical squamous intraepithelial lesion; 14. Cervical glandular intraepithelial neoplasia; 15. Prostatitis; 16. Prostatic nodule; 17. Varicocele; 18. Prostatic calcification; 19. Prostatic atrophy |

**D、既往检查史/D. Past Medical Examination History**

| **Chinese version** | **English version** |
| --- | --- |
| D1.1、最近一年内您是否进行过 C13 或 C14 尿素呼气试验（幽门螺旋杆菌）检测？ 1．是（跳转至 D1.1.1）；2．否（跳转至 D1.2） | D1.1. In the past year, have you undergone a C13 or C14 urea breath test (Helicobacter pylori)? 1. Yes (go to D1.1.1); 2. No (go to D1.2) |
| D1.1.1、如果您进行过 C13 或 C14 尿素呼气试验（幽门螺旋杆菌）检测，那么结果为？ 1．阴性；2．阳性；3．不清楚 | D1.1.1. If yes, what was the result? 1. Negative; 2. Positive; 3. Not sure |
| D1.2、最近一年内您是否进行过粪便潜血试验（大便）检测？ 1．是（跳转至 D1.2.1）；2．否（跳转至 D1.3） | D1.2. In the past year, have you had a fecal occult blood test? 1. Yes (go to D1.2.1); 2. No (go to D1.3) |
| D1.2.1、如果您进行过粪便潜血试验（大便）检测，那么结果为？ 1．阴性；2．阳性；3．不清楚 | D1.2.1. If yes, what was the result? 1. Negative; 2. Positive; 3. Not sure |
| D1.3、最近一年内您是否进行过 EB 病毒检测？ 1．是（跳转至 D1.3.1）；2．否（跳转至 D1.4） | D1.3. In the past year, have you been tested for Epstein–Barr virus (EBV)? 1. Yes (go to D1.3.1); 2. No (go to D1.4) |
| D1.3.1、如果您进行过 EB 病毒检测，那么结果为？ 1．阴性；2．阳性；3．不清楚 | D1.3.1. If yes, what was the result? 1. Negative; 2. Positive; 3. Not sure |
| D1.4、最近一年内您是否进行过人乳头瘤病毒（HPV）核酸检测？ 1．是（跳转至 D1.4.1）；2．否（跳转至 D1.5） | D1.4. In the past year, have you undergone HPV nucleic acid testing? 1. Yes (go to D1.4.1); 2. No (go to D1.5) |
| D1.4.1、如果您进行过人乳头瘤病毒（HPV）核酸检测，那么结果为？ 1．阴性；2．阳性；3．不清楚 | D1.4.1. If yes, what was the result? 1. Negative; 2. Positive; 3. Not sure |
| D1.5、您在儿童期时是否有头颈部放射史？（如进行颈部 CT、胸椎 X 线等检查） 1．是；2．否 | D1.5. Did you have head/neck radiation exposure during childhood? (e.g., neck CT, thoracic spine X-ray) 1. Yes; 2. No |
| D1.6、您是否曾经不幸罹患癌症？ 1．是（跳转至 D1.6.1）；2．否（跳转至 D2 家族史） | D1.6. Have you ever been diagnosed with cancer? 1. Yes (go to D1.6.1); 2. No (go to D2) |
| D1.6.1、您曾经不幸罹患癌症，请问是哪种癌症？（多选） 1．肺癌；2．乳腺癌；3．甲状腺癌；4．肠癌；5．胃癌；6．宫颈癌；7．肝癌；8．结（直）肠癌；9．前列腺癌；10．鼻咽癌；11．食管癌；12．子宫癌；13．肾癌；14．胰腺癌；15．膀胱癌；16．卵巢癌；17．其他，请填写______ | D1.6.1. If yes, which cancer type(s)? (multiple choice) 1. Lung; 2. Breast; 3. Thyroid; 4. Intestinal; 5. Gastric; 6. Cervical; 7. Liver; 8. Colorectal; 9. Prostate; 10. Nasopharyngeal; 11. Esophageal; 12. Uterine; 13. Renal; 14. Pancreatic; 15. Bladder; 16. Ovarian; 17. Other, please specify ______ |
| D2.1、您的一级亲属中（父母、子女、兄弟姐妹），是否有人不幸罹患癌症？ 1．是（跳转至 D2.1.1）；2．否（跳转至 D3 肝脏病史） | D2.1. Have any of your first-degree relatives (parents, children, siblings) been diagnosed with cancer? 1. Yes (go to D2.1.1); 2. No (go to D3) |
| D2.1.1、如果您的一级亲属中，有人不幸罹患癌症，请问是哪位亲属？（多选） 1．父亲；2．母亲；3．儿子；4．女儿；5．兄弟；6．姐妹 | D2.1.1. If yes, which relative(s)? (multiple choice) 1. Father; 2. Mother; 3. Son; 4. Daughter; 5. Brother; 6. Sister |
| D2.1.2、如果您的一级亲属中，有人不幸罹患癌症，请问是哪种癌症？（多选） 1．肺癌；2．乳腺癌；3．甲状腺癌；4．肠癌；5．胃癌；6．宫颈癌；7．肝癌；8．结（直）肠癌；9．前列腺癌；10．鼻咽癌；11．食管癌；12．子宫癌；13．肾癌；14．胰腺癌；15．膀胱癌；16．卵巢癌；17．其他，请填写______ | D2.1.2. If yes, which cancer type(s)? (multiple choice) 1. Lung; 2. Breast; 3. Thyroid; 4. Intestinal; 5. Gastric; 6. Cervical; 7. Liver; 8. Colorectal; 9. Prostate; 10. Nasopharyngeal; 11. Esophageal; 12. Uterine; 13. Renal; 14. Pancreatic; 15. Bladder; 16. Ovarian; 17. Other, please specify ______ |
| D3.1、您的一级亲属中（父母、子女、兄弟姐妹），是否有人曾患有乙型肝炎？ 1．是；2．否 | D3.1. Have any first-degree relatives had hepatitis B? 1. Yes; 2. No |
| D3.2、您的一级亲属中（父母、子女、兄弟姐妹），是否有人曾患有肝硬化？ 1．是；2．否 | D3.2. Have any first-degree relatives had liver cirrhosis? 1. Yes; 2. No |

**男性受访者请停止问卷，女性受访者请继续填写。
Male respondents, please stop here; female respondents, please continue.**

**E、女性生理和生育（仅女性填写）/E. Female physiology and reproduction (for women only)**

| **Chinese version** | **English version** |
| --- | --- |
| E.1、您的首次月经年龄是？请填写 ______ 岁 | E.1. At what age did you have your first menstruation? Please fill in ______ years. |
| E.2、您是否已有性生活？ 1．是（跳转至 E.3）；2．否（跳转至 E.5） | E.2. Have you ever had sexual activity? 1. Yes (go to E.3); 2. No (go to E.5) |
| E.3、您是否有生育史？ 1．是（跳转至 E.3.1）；2．否（跳转至 E.4） | E.3. Have you ever given birth? 1. Yes (go to E.3.1); 2. No (go to E.4) |
| E.3.1、累计顺产胎数为（指分娩次数而非孩子数量） ______ 胎，剖腹产胎数为 ______ 胎。 | E.3.1. Total number of vaginal deliveries (refers to number of deliveries, not number of children): ______; Caesarean deliveries: ______. |
| E.3.2、首次分娩时，您的年龄是 ______ 岁。 | E.3.2. How old were you at your first delivery? ______ years. |
| E.4、您是否有哺乳史？ 1．是（跳转至 E.4.1）；2．否（跳转至 E.5） | E.4. Have you ever breastfed? 1. Yes (go to E.4.1); 2. No (go to E.5) |
| E.4.1、累计哺乳月数为（不足 1 个月按 1 个月计） ______ 月 | E.4.1. Total months of breastfeeding (count less than one month as one month): ______ months. |
| E.5、您是否已绝经？ 1．是（跳转至 E.5.1）；2．否（跳转至 E.6） | E.5. Have you reached menopause? 1. Yes (go to E.5.1); 2. No (go to E.6) |
| E.5.1、您的绝经年龄是？请填写 ______ 岁 | E.5.1. At what age did you reach menopause? Please fill in ______ years. |
| E.6、您是否使用激素替代治疗达 6 个月及以上？ 1．是，仅雌激素（如更宝芬、补佳乐、协坤、维尼安、更乐、倍美力、得美素、欧适可、松奇、康美华、尼尔雌醇等）；2．是，雌孕激素联合（如诺康律、诺更宁、克龄蒙、倍美安、倍美盈等）；3．否 | E.6. Have you used hormone replacement therapy (HRT) for six months or longer? 1. Yes, oestrogen only (e.g., Geng Bao Fen, Progynova, Xie Kun, Weinian, Geng Le, Bemily, Demisu, Ousike, Songqi, Kangmeihua, Nilestradiol, etc.); 2. Yes, combined oestrogen–progestogen (e.g., Nokanglü, Nuo Gengning, Kailingmeng, Bemian, Bemeiying, etc.); 3. No |
| E.7、您是否有乳腺活检史或乳腺良性疾病手术史？（经正规医疗机构诊断） 1．是；2．否 | E.7. Have you ever had a breast biopsy or surgery for benign breast disease (diagnosed by a certified medical institution)? 1. Yes; 2. No |
| E.8、【乳腺】您是否曾经确诊以下疾病？（经正规医疗机构诊断）（多选） 1．否；2．乳腺外周型乳头状瘤伴不典型增生；3．乳腺纤维瘤；4．乳腺炎；5．乳腺小叶不典型增生；6．乳腺导管不典型增生；7．乳腺囊肿；8．乳腺小叶瘤变；9．其他，请填写______ | E.8. [Breast] Have you ever been diagnosed with any of the following conditions by a certified medical institution? (multiple choice) 1. No; 2. Peripheral breast papilloma with atypical hyperplasia; 3. Breast fibroadenoma; 4. Mastitis; 5. Atypical lobular hyperplasia of the breast; 6. Atypical ductal hyperplasia of the breast; 7. Breast cyst; 8. Lobular neoplasia of the breast; 9. Other, please specify ______ |

**本问卷到此结束，感谢您的参与
This is the end of the questionnaire. Thank you for your participation.**

**本问卷最终解释权属珠海市附加补充医疗保险“大爱无疆”项目所有
The final right of interpretation of this questionnaire belongs to the “Great Love Without Boundaries” supplementary medical insurance programme of Zhuhai.**
